# The Effect of Age on Electronic Health Literacy: Mixed-Method Study

**DOI:** 10.2196/11480

**Published:** 2019-04-21

**Authors:** Wan-Chen Hsu

**Affiliations:** 1 Center for Teaching and Learning Development National Kaohsiung University of Science and Technology Kaohsiung Taiwan

**Keywords:** eHealth literacy, intergenerational relations, traditional college students, older adult students, mixed method

## Abstract

**Background:**

The world’s internet penetration rate is increasing yearly; approximately 25% of the world’s population are internet users. In Asia, Taiwan has the fifth highest internet usage, and has an internet penetration rate higher than the world average. Electronic health (eHealth) literacy is the ability to read, understand, and utilize Web health information. eHealth literacy is gaining attention worldwide.

**Objective:**

This study aimed compare the differences in eHealth literacy between traditional college students (aged between 18 and 22 years) and older adult students (aged between 55 and 72 years). It also summarizes the experiences and performances of these 2 groups in terms of searching online health-related information.

**Methods:**

A mixed-method approach was used, including questionnaire surveys and interviews. A total of 208 respondents were interviewed: 65 traditional college students (31.3%) and 143 older adult students (68.7%). The results of the interviews were used to compare the eHealth literacy scores of the 2 groups.

**Results:**

There were significant differences in the overall eHealth literacy scores (*t*_207_=2.98; *P*=.001) and the functional eHealth literacy dimension (*t*_207_=12.17; *P*<.001). The findings showed a significant gap in eHealth literacy between the 2 groups. Most participants believed that online health information could be largely read and understood. However, they were skeptical about the quality of the information and noted that it consisted of either subjective judgments or objective standards.

**Conclusions:**

Traditional college students preferred esthetically pleasing health information, whereas older adult students focused on its promotion. Furthermore, the first group often used websites for solving health problems, whereas the second group forwarded health information through communication software.

## Introduction

### Increased Internet Usage and the Issue of Electronic Health Literacy

Electronic health (eHealth) literacy is gaining increasing attention worldwide. Individuals with eHealth literacy have better health capital and can further promote the overall health and competitiveness of their countries. In 2017, the Taiwan Broadband Internet Usage Survey reported that results from 3153 valid sample analyses showed that individuals were accustomed to having an internet access rate of over 83%. Of these individuals, 84.7% agreed that “the use of the Internet has improved the quality of your life.” However, 55.9% disagreed with the statement that “the use of the Internet can increase your trust in information” [1]. Thus, even if individuals possessed little knowledge of eHealth information, it was still possible for them to take appropriate action.

### Different Age Groups Exhibit Unique Electronic Health Literacy Performances

Given the popularity of the internet, research has shifted focus toward the relevance of health literacy through the internet; as a result, “eHealth Literacy,” as a field of research, has gradually received increased attention [2]. Eng [3] argues that *eHealth literacy* refers to the use of the internet to access information to improve or promote health, whereas Norman and Skinner [4] further suggest that *eHealth literacy* refers to the use of the internet to seek, understand, and evaluate health information and to use this information to address health problems.

Health literacy includes 3 dimensions [5], and it is the main constituent of eHealth literacy [2]. Chiang et al [6], using the definition of health literacy, divided the 3 dimensions into functional eHealth literacy, interactive eHealth literacy, and critical eHealth literacy. The first layer is *functionality*, which refers to the essential ability to read health information. The second layer is *interaction*, which refers to more advanced knowledge of the choices available in health information, including knowledge required to understand, integrate, and use that information and knowledge of a supportive, interactive environment that provides health information and other skills. The third layer is *criticality*, which is a more in-depth analysis of health information and involves both criticisms of the information and its application to health and the response to that criticism, resulting in better control over living conditions.

### Related Research on Electronic Health Literacy

eHealth literacy has the potential to positively support consumers’ health empowerment [7]. The Integrative Model of eHealth Use claims that macro-level disparities in social structure are connected to health disparities that arise because of micro-level factors such as eHealth literacy, motivation, and ability [2]. Few studies have explored the associations among individual factors such as gender, age, and college major in relation to eHealth literacy [8,9], as cross-group comparisons have yet to be investigated empirically. In Taiwan, which currently has the fifth-highest internet usage in Asia and has an internet penetration rate of 65.90% [10], college students constitute 1 of the groups that access internet health information more frequently. The proportion of older people using information and communication technology appears to be lower than that of other age groups, making it difficult to breach this digital wall. Currently, there are nearly 300 active aging learning centers in Taiwan, which are the leading institutions of learning for retired older adults. They are designed to receive students over the age of 55 years [11]. This leads to the question examined in this study: Does eHealth literacy overcome the generation gap? Specifically, is there a difference between eHealth literacy among traditional college students and older adult students? To answer these questions, this study examined the differences in eHealth literacy among older adult students (people over the age of 55 years) and among traditional college students (aged from 18 to 22 years, thus within the conventional age range for university undergraduates in Taiwan) to gain an in-depth understanding of the differences that exist across age groups.

Eysenbach and Köhler [12] explored the use of health information by internet users and found that participants’ assessment of the quality of online health information included the authority, appearance, and layout of the source; advertising; readability; the presence (or absence) of links to other websites; website holder photos; contact boxes; website certification; content updates; quality badges; or other professional group support. There is limited literature on the experience of college students using online health information. Within that literature, studies have shown that traditional college students possess functional and interactive health literacy levels and seem to underperform at a higher level of critical health literacy. Relevant studies have shown that college students are confident that they can find, read, and understand online health information [8,13]. However, a high proportion of these students are less assured in their ability to discriminate between high- and low-quality health resources [13].

Compared with younger adults, older adults had less confidence in eHealth resources, information-seeking skills, and the ability to evaluate and act upon online health information [14]. Lee et al [15] note that increased age is a factor that is frequently associated with decreased levels of eHealth literacy. Older adults with chronic health conditions and those with lower levels of eHealth literacy were prone to unmet navigational needs, experiencing difficulties in finding online health information, and being less assured in their searching abilities [15]. An investigation of internet skills also found that older adults sometimes experienced problems when completing tasks that called upon operational and formal internet skills [16]. This included difficulties in understanding orientation within a website and identifying and using the browser address bar. A survey that addressed the health information–seeking behaviors of baby boomers and older adults found that an increase in age corresponded to a decrease in eHealth literacy scores [17]. However, in contrast to other research studies, Lee et al [15] found that the respondents were mostly confident regarding their ability to find and use internet-based health resources, although they were less confident in their ability to differentiate between high- and low-quality resources. The more relevant generational differences were based on quantitative research. The novelty of this study is that it takes into account both quantitative and qualitative research methods in coming to an understanding of the prevalence of eHealth literacy among participants of different generations.

In Taiwan, nearly half of traditional college students use internet health information as a conduit for self-diagnosis [18]. Moreover, in April 2018, the proportion of people over the age of 65 years reached 14% in Taiwan’s population structure. Therefore, Taiwan has officially become an “aged society” [19] that pays attention to the current situation of traditional college students and elders in eHealth literacy and is more concerned with the overall competitiveness of the country in the future.

Taiwan has made improving the health literacy of adults a cornerstone of national health policy. At present, the research on health literacy–related topics mostly focuses on the preparation of measurement tools [9,20,21] and the current lack of intergenerational differences in adult eHealth literacy. This study used a mixed-method perspective to explore the eHealth literacy of different groups of traditional college students and older adult students. Complementing the understanding of the current situation on eHealth literacy is the importance of exploring health literacy as a topic of national policy and the bridging of gaps in the research literature on the subject. Information literacy and health literacy are important connotations of eHealth literacy [4]. In study 1, *a quantitative analysis is performed to explore eHealth literacy between different age groups*. eHealth literacy has been investigated by the electronic Health Literacy Scale (eHLS) and clearly represented in participants’ eHLS scores. These scores correspond to self-reported ability to find and evaluate online health information. Thus, study 2 focuses on participants’ information-seeking behaviors, patterns, and preferences and on their skills in detecting online health information. To examine this issue further, this study also summarized the online health information–seeking behaviors, patterns, and preferences of both traditional college students and older adult students concerning skills, experiences, and performances. The purpose of this study was to compare the eHealth literacy scores of both groups of students. Moreover, by collecting the online health information of these groups through interviews and then summarizing this information, this study sought to read and understand what constitutes relevant experience for each group.

## Methods

### Overview

This study uses a sequential mixed-method design. Such a design uses qualitative and quantitative research sequentially, depending on the purpose or problem of the study. The purpose is both to attain “complementarity,” such as the rationality of quantitative data in additional sampling, and to further research through qualitative study [22]. Accordingly, this study was carried out in 2 phases. The first phase, comprising the *quantitative* part of the study, was mainly used to screen respondents. The second phase consisted of an interview to collect the respondents’ eHealth literacy data, which were analyzed *qualitatively*.

### Recruitment

In this mixed-method study, the research process was divided into 2 phases. First, an eHealth literacy instrument (a questionnaire) was used to investigate the participants’ current situation. In this first stage, 2 classes of traditional college students in the general education program were assessed. Moreover, 3 classes of older adult students (aged 55 years or above) who were enrolled in a university-affiliated, formal, unaccredited, voluntary, lifelong learning program participated in this study. The older adult students who participated had at least an elementary school education; some studied at the senior high school level, although not all attained a diploma and none had a college degree. Data were collected from an urban university in Taiwan. Before the study, the program was reviewed and approved by the university’s institutional review board (ethics committee). Of the 208 respondents, 65 (31.2%) were traditional college students (aged between 18 and 22 years) and 143 (68.7%) were older adult students (aged from 55 to 72 years).

The answers to the questionnaire were evaluated to select prospective respondents as the second phase interviewees. The participants were chosen from a voluntary sample (see Table 1). This resulted in the selection of 5 traditional college students and 5 older adult students as the interviewees. Interviews took place from January 2017 to February 2017 and lasted for about 1 hour.

**Table 1:** Demographic data on the interviewees.

Interviewees, gender		Age (years)	Educational level
**Traditional college student**			
	Male	20	Studying at university (sophomore, the second year of college)
	Male	20	Studying at university (sophomore, the second year of college)
	Male	19	Studying at university (freshman, the first year of college)
	Female	21	Studying at university (junior, the third year of college)
	Female	20	Studying at university (sophomore, the second year of college)
**Older adult student**			
	Male	57	Senior high school
	Male	62	Junior high school
	Female	70	Junior high school
	Female	59	Senior high school
	Female	68	Elementary school

### Instrument

The eHLS measures a student’s ability to seek, find, understand, and evaluate health information from electronic sources and to apply this knowledge to address or solve a health problem [4,9,14]. A “gold standard” eHealth literacy instrument, which health researchers have recommended as an account of the social nature of eHealth, is being discussed by health professionals at the time of writing of this manuscript [23]. The 12-item eHLS is an instrument used to measure eHealth literacy among adult Taiwanese individuals. It was developed by Chiang et al [6], who surveyed a representative sample of college students between March 2014 and May 2014, obtaining 455 valid responses. The reliability of the individual eHLS items ranged from .36 to .74. Standardized factor loading ranged from .60 to .86 (*P*<.001). Composite reliability ranged from .75 to .84, and the average variance extracted for each dimension ranged from .50 to .52. The indicators demonstrated a good fit for the measurement model. The scale includes functional (3 items, Cronbach alpha=.70), interactive (4 items, Cronbach alpha=.77), and critical (5 items, Cronbach alpha=.83) eHealth literacy dimensions. Its internal structure and external validity are considered acceptable. Respondents were asked to select the most accurate answer to describe their eHealth literacy level on a 5-point Likert scale, ranging from 1=strongly disagree to 5=strongly agree. Cronbach alpha of the overall scale was .84 (Multimedia Appendix 1).

One of the aims of this study was to try to capture the experience of the use of online health information for college students and older adult students. To do so, this study examined the participants’ health literacy levels based on Nutbeam’s [5] 3 levels of health literacy and definitions of eHealth literacy from Hsu et al (2011) [9] and Hsu et al (2014) [8]. Semistructured interviews were conducted to collect data. The interview outline included 4 items (see Table 2).

### Data Analysis

Descriptive statistics and *t* tests were conducted to understand the effect of age on eHealth literacy. In addition, in conducting qualitative data analysis [24], the researcher conceptualized and developed a protocol to ensure open coding. Next, the analysis applied the concept of higher extraction level to “axial coding,” which arranges concepts of similar content together into a class. The axial coding was then used, through classification, comparison, and induction, to analyze the subcategory and the main category together. To facilitate the classification and analysis of research data, the first column of Table 3 identified the participants, that is, A (traditional college student) and B (older university student). The second column identified the interview number. The third column identified an encoded serial number (eg, A-1-2).

**Table 2:** Interview guide items.

Focus point, open questions		The concept of health literacy
**Access to online health information** [7]		
	What kind of online health information are you more interested in?	Basic ability to read health information (*functional literacy*)
	What kind of source for online health information are you more interested in?	Knowledge of a supportive, interactive environment that provides health information (*interactive literacy*)
**Literacy experience of online health information** [3]		
	How would you evaluate your internet health information reading and implementation experiences?	Basic ability to read and use health information (*functional* literacy and *interactive literacy*)
	How do you assess the accuracy of health information? What is the assessment principle?	Both criticism of health information and its application to one’s health *and* the response to that criticism, resulting in better control over living conditions (*critical literacy*)

**Table 3:** Coding and categorization examples.

Main category, subcategories, axial coding			Open coding
**The experience of participants in obtaining online health information differed by group**			
	**Access devices**		
		College students access most online health information from Web pages [A-2-64]	We college students retrieved most online health information from Web pages. [A-5-31] The most commonly used Web pages or websites are the occasional Yahoo News health section. [A-2-64]
		Older adult students often obtained online health information through communication software [B-5-11]	We have a Line group in the class; every day we share messages. [B-5-11] Computers are used less now, as mobile phones are the most convenient. We often use communication software to share health information. [B-4-15]
	**Access types of online health information**		
		Traditional college students	At University, my friends always pay special attention to their appearance, so I pay special attention to my looks. [A-1-28]
		Older adult students	Since retirement, I pay special attention to diet and health issues such as exercise and fitness It is good for health. [B-3-5]

The interview addressed the issue of participants’ experiences in accessing online health information and focused on the concept of health literacy. In the process of data analysis, an academic peer was invited to use the code and test its relevance to meet the consistency and reliability requirements of qualitative research.

The purpose of this study was to understand the ways that subjects perceive their experiences. Therefore, the researcher played the role of “listener” in the interview process and did not make value judgments while conducting the interviews. Coding and categorization examples are shown in Table 3.

## Results

### Study 1—Quantitative Analysis of Electronic Health Literacy: Electronic Health Literacy Between Age Groups

There was a significant difference in the overall eHealth literacy scores between traditional college students and older adult students (*t*_207_=2.98; *P*=.001), with the overall scores of traditional college students (mean 43.78) being higher than those of the older adults (mean 40.93). In the functional eHealth literacy dimension (*t*_207_=12.17; *P*<.001), the traditional college students’ scores (mean 11.43) were higher than those of the older adults (mean 8.08). However, there was no significant difference between interactive and critical eHealth literacy (see Table 4). In addition, in this study, we found a difference between age groups; functional eHealth literacy was higher than interactive and critical literacies for traditional college students (*F*=101.28; *P*<.001) and older adult students (*F*=373.24; *P*<.001).

### Study 2—Qualitative Analysis of Electronic Health Literacy: Participants’ Experiences and Performance of Reading Online Health Information

#### First Result: The Experience of Interviewees in Obtaining Online Health Information Differed Between Generations

The initial motivation for college students to access online health information was to meet the needs of beauty, weight loss, fitness, and so on:

Before in high school, I had a face full of acne and now have pockmarks. Now, at University, my friends always pay special attention to their appearance, so I pay special attention to my beauty.

A-5-31

Girls always want to have a good body shape...You may see someone post on the Internet about how they succeeded in losing weight. When we see a successful experience in which someone loses weight quickly, no matter whether it is true or not, we want to learn from them.

A-3-28

**Table 4:** Electronic Heath Literacy Scale means, SDs, and *t* tests.

Factor	Traditional college students (N=65), mean (SD)	Older adult students^,^ (N=143), mean (SD)	The assumption of equal variances	*P* value	*t* test	*P* value
Functional electronic health literacy	11.43 (1.94)	8.08 (1.56)	3.706	.06	12.17 (206)	<.001
Interactive electronic health literacy	14.50 (2.67)	14.60 (2.52)	0.100	.75	−0.263 (206)	.79
Critical electronic health literacy	17.81 (3.74)	18.18 (3.28)	3.214	.08	−0.71 (206)	.48
Electronic health literacy	43.78 (6.68)	40.93 (5.10)	3.711	.06	2.98 (206)	<.001

Furthermore, the online health information retrieval methods of college students can be divided into *fixed* and *nonfixed* Web pages, which were used for 2 different purposes. Fixed habitual behavior of college students was to retrieve health information from static Web pages or magazines and either browse health knowledge or seek suitable skin care products for themselves, rather than solving practical health problems. On the other hand, they also gathered health information from nonfixed pipelines with the aim of solving health problems that were of immediate concern. Often, when respondents were aware of health problems, they would conduct online health information retrieval, looking up information about, for example, acne, weight loss, treatment of colds, gastrointestinal care, medical topics (such as new flu prevention), cancer diet, and so on. Data revealed that respondents searched using search engines and entered keywords but did not use search techniques involving, for example, Boolean logic. Respondents often looked only at the first search result. If there were many websites retrieved, there was a greater possibility that respondents would find a Web page with an appropriate answer or a Web page that was familiar to them. If there were links to other Web pages, they would click those links. However, if the health message of the interviewee was based on personal experience, there were limitations to his or her search for this kind of individual-oriented experience. Such internet health information was offered only as a reference, and it had little influence on actual implementation:

The most commonly used site is the occasional Yahoo news health section, on things like healthcare, healthy food, health exercises, and clicking on links to help with acupuncture points and the like, such as ah-shi acupuncture points for the eye.

A-2-64

I often use Yahoo or Google and get many results from forums...just by looking at a forum or seeing someone share an experience. Message-board posts can only be used as reference points that are not easy for individuals to adopt. This is because everyone has a different living environment and because the retrieved information cannot be used for all situations.

A-4-73

Respondents were most interested in reading online health information. When browsing health information, most of the information available online was not sufficiently clear or too specialized. Moreover, most respondents asserted that they understood the health information on the internet, but some proper nouns, foreign language terms, and various ways in which data were presented made it difficult for some respondents to understand and learn from adverse outcomes:

Most of the online health information is easy to understand and does not appear [for example] like a book that is written very professionally and difficult to read. Getting information from the Internet is very convenient, [because] you can quickly know the information and it will not be difficult [to understand].

A-1-41

Furthermore, the criteria for assessing the quality of online health information can be divided into 2 categories: subjective judgment and objective form. *Subjective judgment* is based on the cognitive judgment of the respondents; thus, they use their preknowledge to evaluate health information. Some would take the initiative by seeking others’ advice to confirm the quality of online information, specifically by cross-validating through different Web pages or asking professionals (eg, doctors, pharmacists, medical friends, and relatives) directly to arrive at a more credible conclusion:

There is some very obviously illogical [information that] I will not go to, at least with the mind to question it. For example, burn soy sauce, or wipe pepper on your skin for weight loss, but you would think that this will only hurt your skin.

A-5-35

On the other hand, the *objective form* refers to the quality assessment of respondents from the external mode of online health information. In addition, some of the respondents would direct their attention toward the sources used by the specific internet source, particularly focusing on “update time,” “certification mark,” “whether it was an official website,” “the number of visitors,” “whether there are small ads,” and so on:

Reading numbers, small ads...If the site appears with too many small ads, that is, with commercial activity, [with an intent] to sell some things, I will not accept it.

A-3-98

I will check information from an accredited institution...[If called to choose] [b]etween information available for 2005 and 2009, I would rather believe information from 2009, because it is relatively new. The content is really a relatively large problem; I will pay attention to the source...If it is just the Central Research Institute meeting with a doctor, I will ask that day to see that...that perspective?

A-2-91

Online health information used by older adult students was mostly concerned with “diet and nutrition,” “health and wellness,” and “exercise and fitness”:

Before, when I was working, I was often busy when I ate, so I did not pay attention to nutrition, had no time to exercise, causing me to be physically ill often, and also susceptible to catching a cold, and now, after retirement, I pay special attention to diet and health issues such as exercise and fitness. I think exercise [is important] for your health.

B-3-5

Besides, older adult participants had access to a communications software group, enabling them to access online health information via group sharing:

We have a Line group in the class, [and] every day we share messages. Last month a student was sick and suddenly died. We are already into old age, so we all attach great importance to health problems, and if we do not maintain our health, we will soon meet God. So, we have a bunch of health messages every day to share with each other, every day mobile phone messages to forward.

B-5-11

I am now sixty years old, at the age when I begin to face what old, sick, dead, and living must go through, when you hear friends around who get sick and then survive, they become more self-alert. Computers are used less now, as mobile phones are most convenient, I now have time to draw...We often use communication software to share health information to friends and relatives, in the hope that people can keep healthy.

B-4-15

The above data indicated that the majority of older adult students had retired from work and had more time to pay attention to their health, focusing more on their “diet and nutrition,” “health and wellness,” and “exercise and fitness.” Their primary source of information was mobile communication software through which they shared health messages.

#### Second Result

Most of the respondents believed that they had no problem with reading comprehension. However, most of the older adult students observed that they had lower critical ability and difficulty distinguishing correct information from incorrect information. Therefore, they believed that most health information was not very reliable as a reference:

There are a lot of opportunities to share health information, [and] reading comprehension is not a problem. I am also interested in the content, but some of the information overlaps, and I cannot evaluate the accuracy, so the reference value is reduced. Also, I have to follow the practice of health information. But the effect [once the information is applied] is not as favorable as the health information itself, so for these messages, after reading the reference, the effect is more difficult to control.

B-3-45

Older adult students primarily based their assessment of information accuracy on both subjective judgment (such as individual experience or previous knowledge) and objective standards (such as sources, publication date, or professional authorship). These respondents used implicit perceptions of their own experiences and subjective judgments as well as explicit perceptions, such as information from experts and cross-validation of information from credible institutions:

Online health information needs to be cross-verified for correctness through different channels, such as seeking formal medical information medical information or asking a medical professional.

B-1-71

## Discussion

### Electronic Health Literacy Performances Vary Across Generations

In the overall assessment of eHealth literacy, traditional college students scored higher than older adult students. The results indicate that eHealth literacy varies across generations. eHealth literacy has multiple levels [6]: the first layer, *functionality*, is the basic reading ability; the second layer, *interaction*, is advanced knowledge; and the third layer, *criticality*, is more in-depth analytical ability to judge [25]. This may be explained by the concept of cognitive dimensions, as both functional and critical eHealth literacy involve higher levels of ability that require more extended periods of cognitive training to develop. In this study, young college students were more educated than older adult students. Therefore, it was likely that the level of educational attainment for these college students would be high, and, consequently, eHealth literacy levels would also be high [26]. Accordingly, college students had higher scores in eHealth than older adult students.

### Individual Experience of Accessing Online Health Information

This study found that traditional college students and older adult students accessed online health information differently. Young adult college students accessed online information from websites and focused mostly on “beauty,” “weight loss,” and “fitness.” These topics typically concern physical appearance. Older adult students accessed online health information generally through communication software, enabling them to share more health information. The health information older adult students tended to access included information about diet and nutrition, health and wellness, and exercise and fitness.

Moreover, most of the study participants had expressed the belief that the majority of online health information could be read and understood. However, when evaluating the quality of information, they were generally doubtful. Participants pointed out that the quality of online health information is divided into subjective judgment and standard objective information. However, some participants in each group suggested that, in the era of information explosion, it is not easy to choose and determine the accuracy of the information. Thus, the participants tended to take a skeptical attitude if they were personally involved and would then find additional resources to verify through multiparty comparison to enrich their knowledge and understanding of the topic. This finding is similar to the findings in the study by Hsu et al [8]. This may be explained by the Uncertainty Management Theory (UMT) [27]. A prominent communication uncertainty framework has been applied to appraise the associations between online health information seeking and uncertainty management [28,29]. A central tenet of UMT proposes that uncertainty is not necessarily a negative or positive experience; instead, an individual will appraise the meaning of uncertainty, and the resulting emotional response will determine whether the uncertainty is evaluated as negative, positive, or neutral. The uncertainty evaluation will influence an individual’s behaviors in managing his or her uncertainty. For example, individuals for whom uncertainty is an undesirable or negative state may seek health information to augment their knowledge and thereby lessen their state of uncertainty [30].

Furthermore, in study 1, functional eHealth literacy scores were different for traditional college students and older university students, but interactive and critical eHealth literacy scores did not differ. Empirical evidence has shown that an inverse correlation exists between age and eHealth literacy [17,31,32]. In study 2, the experience of how interviewees obtained online health information differed according to generation. Most interviewees could read online health information, but older adult students had less critical ability to do so. Paige et al [14] indicated that, compared with younger adults, older adults had less ability, possibly owing to cognitive degradation, to evaluate and act upon online health information. Bodie and Dutta [2] pointed out that antecedents such as personality and educational background are the factors that influence the individual’s eHealth literacy. The interviewees in this study received different modes of cognitive training, which might be responsible for the difference in the results and also highlights the need for eHealth literacy to be introduced into education.

### Limitations

This study has several limitations. First, the study was performed under trial conditions in which it was expected that the participants would answer hypothetical questions that were not clear and did not directly address the participants’ actual physical health concerns. Second, this study found that both younger college students and older adult students were capable of reading and understanding online health information to some extent. The participants did have basic eHealth literacy. However, both groups experienced difficulties in recognizing correct health information online. Given that we did not observe the highest level of critical eHealth literacy, the data collected through our investigation may not have been specific enough. Furthermore, the interviewees constituted a voluntary sample of 5 participants, which likely affected the generalizability of the results.

This study found that functional eHealth knowledge varies across age groups, unlike interactive and critical scores. Higher levels of eHealth knowledge are more difficult to cultivate and evaluate [5,6,8]. Given that most people do not have the ability to attain higher-level health literacy, there were no differences between groups. However, some studies have pointed out that older adults had less confidence in eHealth resource awareness, information-seeking skills, and the ability to evaluate and act upon online health information [14]. This study used interviews to understand the participants’ capacity to read, understand, and critically evaluate health information, along with other relevant experience. It collected and analyzed the participants’ self-reported data. However, there is a difference between the online performance of the participants and their real offline experience of eHealth literacies. Future studies could record the actual online behavior of the participants, utilizing a specific observable behavior analysis, and investigate how the subjects demonstrate the 3 levels of eHealth literacy. This would give a better estimation of their actual abilities [26].

### Conclusions

This study explored the effect of age on eHealth literacy in the hope that the findings will stimulate further debate about how a health education framework can be translated into practical approaches and contribute to the further refinement of the eHealth literacy concept. In the eHealth literature, this is the first study to explore whether group differences exist.

The findings showed that there are gaps in eHealth literacy between traditional college students and older adult students. Therefore, we recommend that the education system strengthens the functional and critical eHealth literacy of both groups. These age disparities have been attributed to older adults’ unique health needs (eg, “diet and nutrition,” “health and wellness,” and “exercise and fitness”) as compared with younger adults [33] and to more specialized health concerns (eg, beauty, weight loss, and fitness) of college students.

Due to different personal eHealth literacy experiences, to develop specific strategies for promoting individualized health literacy, it is necessary to know about the current health concerns of different individuals not only to design eHealth learning programs but also to empower individuals in planning them. The study observed that participants, when faced with challenging and uncertain health situations, employed various strategies to reduce ambiguity about a health-related condition. Furthermore, based on Nutbeam’s original conceptualization, Paige et al [34] proposed the Transactional Model of eHealth Literacy, which has theoretical underpinnings in transactional communication literature, and adds a fourth level of “translational eHealth literacy.” This is the highest cognitive level of eHealth literacy, and it is informed and builds upon lower-level eHealth literacy dimensions (ie, critical, communicative, and functional). Future studies could investigate how the internet may provide a useful and valuable channel for health information to consumers who wish to utilize information strategies for managing health-related uncertainty.

Moreover, literacy, numeracy, decision-making, and reasoning skills may be needed for the critical evaluation of the retrieved information by each group of students [26]. These skills reflect more critical and translational elements of online information processing, inviting future study. The study summarizes online health information experiences and performances of traditional college students and older adult students. As data collection may involve participants’ subjective perception of their abilities, it is not easy to determine skill-based literacies. Future research is needed to explore this issue.

## Appendix

Multimedia Appendix 1 The eHealth Literacy Scale.

## Abbreviations

eHealth: electronic health
eHLS: electronic Health Literacy Scale
UMT: Uncertainty Management Theory
