# Supplementary material for: The Effect of Age on Electronic Health Literacy: Mixed-Method Study
Source: JMIR Hum Factors. 2019 Apr 21;6(2):e11480. doi: 10.2196/11480 (PMC6526685; doi:10.2196/11480)
Supplement: Multimedia Appendix 1 [file humanfactors_v6i2e11480_app1.pdf]

Appendix: The eHealth Literacy Scale (eHLS)

Functional eHealth literacy	1. I think the mathematical formulas of the Internet health information (ex. the algorithm on heat consumption) are difficult to calculate.<br>2. I feel that the text of the Internet health information is difficult to understand.<br>3. I cannot understand the symbols used in health information, such as BMI (Body Mass Index).
Interactive eHealth literacy	4. I can understand the Internet health information I have received.<br>5. I can select the information I need from the Internet health information.<br>6. I can pay attention to acquiring new information from Internet health information.<br>7. I can find online health information efficiently through search engines.
Critical eHealth literacy	8. When I have questions about health information online, I verify it through other methods.<br>9. I can consult people to discuss questions and make healthy decisions or actions.<br>10. I can check the validity and reliability of my Internet health information.<br>11. I try to find different sources to verify Internet health information.<br>12. I can evaluate whether the Internet health information applies to my situation.
